# The Value of Radiotherapy for Advanced Non-Small Cell Lung Cancer With Oncogene Driver-Mutation

**DOI:** 10.3389/fonc.2022.863715

**Published:** 2022-05-13

**Authors:** Jinfeng Cui, Li Li, Shuanghu Yuan

**Affiliations:** ^1^ Clinical Medical College, Shandong University, Jinan, China; ^2^ Department of Radiation Oncology, Shandong Cancer Hospital and Institute, Shandong First Medical University and Shandong Academy of Medical Sciences, Shandong Cancer Hospital Affiliated to Shandong First Medical University, Jinan, China; ^3^ Department of Radiation Oncology, Shandong Cancer Hospital Affiliated to Shandong University, Jinan, China; ^4^ Department of Radiation Oncology, The Affiliated Cancer Hospital of Zhengzhou University, Zhengzhou, China

**Keywords:** non-small cell lung cancer (NSCLC), oncogene driver-mutated, radiotherapy, targeted therapy, stage IV

## Abstract

Due to the widespread use of tyrosine kinase inhibitors (TKIs), which have largely supplanted cytotoxic chemotherapy as the first-line therapeutic choice for patients with advanced non-small cell lung cancer (NSCLC) who have oncogene driver mutations, advanced NSCLC patients with oncogene driver mutations had much long median survival. However, TKIs’ long-term efficacy is harmed by resistance to them. TKIs proved to have a limited potential to permeate cerebrospinal fluid (CSF) as well. Only a small percentage of plasma levels could be found in CSF at usual doses. Therefore, TKIs monotherapy may have a limited efficacy in individuals with brain metastases. Radiation has been demonstrated to reduce TKIs resistance and disrupt the blood-brain barrier (BBB). Previous trials have shown that local irradiation for bone metastases might improve symptoms, in addition, continuous administration of TKIs combined with radiotherapy was linked with beneficial progression-free survival (PFS) and overall survival (OS) for oligometastasis or bone metastasis NSCLC with oncogene driver mutations. The above implied that radiotherapy combined with targeted therapy may have a synergistic impact in patients with advanced oncogene driver-mutated NSCLC. The objective of this article is to discuss the value of radiotherapy in the treatment of those specific individuals.

## Introduction

Oncogene driver-mutated non-small cell lung cancer (NSCLC) is a distinct entity in thoracic oncology. Tyrosine kinase inhibitors (TKIs) have revolutionized the treatment of stage IV NSCLC with epidermal growth factor receptor (EGFR) mutation and anaplastic lymphoma kinase (ALK) rearrangement (1). As it turns out, molecular targeted medicines are more effective than regular chemotherapy in individuals with NSCLC who have gene abnormalities (2).

Oral TKIs, such as anti-EGFR (gefitinib, erlotinib, afatinib, osimertinib) or anti-ALK (crizotinib, ceritinib, alectinib), are the first-line treatment for patients with stage IV EGFR-mutant or ALK-rearranged NSCLC. When compared to chemotherapy’s progression-free survival (PFS, 5.6-6.9 months) (3, 4) and overall survival (OS, 8-10 months) (5), TKIs provide a significant survival benefit in these patients, with median PFS and OS ranging from 9.2-18.9 months (6, 7) and 27-38.6 months (8, 9), respectively. In addition, in the ALEX study (10), the median PFS of alectinib had reached 34.8 months and the median OS was not reached after 48.2 months of follow-up. Notably, TKI treatment is also associated with fewer adverse events than chemotherapy. Therefore, TKIs have largely supplanted cytotoxic chemotherapy as the first-line therapeutic option for patients with advanced EGFR-mutant or ALK-rearranged NSCLC (11, 12).

However, with first- and second-generation EGFR-TKIs, the majority of patients develop acquired drug resistance between 9-14 months (7), and with third-generation EGFR-TKI, it takes 18.9 months (6).

Patients with EGFR mutations are also more likely to develop brain metastases (BMs). BMs can account for up to 70% of all cases, much exceeding the frequency of BMs in EGFR wild-type individuals (38%) (13). First- and second-generation EGFR-TKIs have relatively low cerebrospinal fluid (CSF) penetration rates, for example, afatinib, gefifitinib, and erlotinib have all been reported to have penetration rates of <1%, 1%-3%, and 3%-6% respectively (14). A larger peak in CSF concentration than the first and second generation EGFR-TKIs, Osimertinib nevertheless has a much lower concentration in CSF (14.4 nM) than the plasma concentration of 555.3 nM (15, 16).

Preclinical studies have demonstrated a synergistic impact between TKIs and radiotherapy, with the most likely interacting mechanisms being the radiosensitizing action of TKIs and the reduction of TKIs resistance by radiotherapy (17–19).

It is well accepted that certain cell cycle phases are more resistant to radiation’s cytotoxic effects than others, with S phase being the most resistant and G2-M being the most sensitive. TKIs and radiation both increase the percentage of tumor cells in the G1 and G2-M phases while decreasing the percentage of cells in the S phase. When combined with radiation, TKIs cause an extra drop in the fraction of cells in the S phase (20, 21). TKIs also can improve the sensitivity of radiotherapy from other aspects: to enhance radiation-induced apoptosis by inhibiting ras-mediated PI3K-AKT pathway; to inhibit the repair of DNA damage after radiotherapy; to reduce cell proliferation and accelerating re-proliferation by inhibiting EGFR pathway; to inhibit the formation of neovascularization and weaken tumor invasion and distant metastasis (20–22).

Additionally, irradiation can damage the BBB’s integrity, and multiple studies have showed that radiation may be an effective way to open the BBB (23–27). The potential advantages of aggressive LCT for patients with no progress after targeted therapy and use of SBRT for oligometastatic focis are that before additional transfer and diffusion occurs, it may delay or prevent the mergence of resistant clones, as shown by the trials that LCT postpones the time to new metastases (28–30). In terms of tumor burden, local radiotherapy can destroy tumor cells in bone metastases, which further reduce the tumor burden on the basis of targeted therapy (31). Therefore, TKIs in conjunction with radiation may be an option for patients with advanced oncogene driver mutation-positive NSCLC. However, the time of radiation and the techniques to be used have not been defined.

In this review, the value of radiotherapy in primary tumor, brain metastasis, bone metastasis and oligometastasis will be expounded.

## Primary Tumor

NSCLC progression patterns were classified into three categories: progression at the primary or metastatic site of illness, progression to new distant sites, and mixed progression (32). As stated by Al-Halabi H et al.’s (32) and Tang Y et al.’s (33) study, about 40% of NSCLC patients advance at the initial site following TKI treatment. A study of 318 patients treated with gefifitinib by Chen M et al. (34) found that 62.34 percent of the patients had an initial failure site in their lungs, which suggests that thoracic radiation (TRT) may be beneficial for this subset of patients.

Patients with EGFR-mutant lung adenocarcinomas were studied by Yen YC et al. (35) in a countrywide, population-based, propensity score-matched cohort study. Treatment with EGFR-TKI alone until tumour progression and patients who responded to EGFR-TKI treatment received TRT for lung tumours afterward were administered to patients in groups 1 (n=1180) and 2 (n=295), respectively. The endpoint was mortality rate among the treatments, the results showed that the mortality rates of the groups 1 and 2 were 40.25% and 31.19% respectively (p=0.0042). TRT for lung tumours in group 2 was linked with a superior OS in both univariate and multivariate Cox regression analysis.

Another complementary study to Yen YC et al.’s (34) research results conducted by Zheng L et al. (36) showed that first-line therapy with concurrent TKI and TRT for patients with advanced NSCLC harboring EGFR mutated provides long-term control of the primary lung lesion, with a 1-year PFS rate of 57.1% and a median PFS of 13 months that are numerically better than those of erlotinib monotherapy (43%, 11 months). However, this study’s sample size is quite small, with only ten individuals included. Thus, a larger sample size is necessary in a multicenter randomised controlled clinical study to demonstrate the efficacy and safety of concurrent EGFR-TKI and TRT in advanced NSCLC.

Studies on the value of TRT for advanced oncogene-driven NSCLC are summarized in **Table 1**.

**Table 1:** Clinical outcomes of TKI alone or combined with TRT for advanced oncogene-driven NSCLC.

study	study type	treatment arm 1	PFS (months)	OS (months)	mortality rate	treatment arm 2	PFS (months)	OS (months)	mortality rate	P value
Yen et al. (35)	retrospective	TKI alone	N	N	40.25%	TKI+TRT	N	N	31.19%	0.0042
Zheng et al. (36)	prospective	–	–	–	–	TKI+TRT	13	N	N	–
Wang et al. (37)	retrospective	TKI alone	10.8	27.8	N	TKI+TSBRT	17.8	36.7	N	P_1 =_ 0.033
Blake-Cerda et al. (38)	prospective	–	–	–	–	TKI+SABR	34.3	N	N	–
Kotek Sedef et al. (39)	retrospective	TKI alone	12	23	N	TKI+TRT	13	33	N	P_1 =_ 0.75 P_2 =_ 0.05

TRT, thoracic radiotherapy; SBRT, thoracic stereotactic body radiotherapy; SABR, stereotactic ablative radiotherapy; N, no mention in the paper; PFS, progression-free survial; OS, overall survival; P_1_, P value of PFS; P_2_, P value of OS.

However, there is no definite conclusion on the timing of radiotherapy for tumors. A study has shown that for patients with relatively low primary tumor burden, time to response (TTR) of responders and time to maximal tumor shrinkage (TTM) of patients with stable disease (SD) may be an excellent opportunity for metastatic patients to combine local treatment. The median TTR was 2 months (95% CI 1.28-2.92 months) for responders, and the median TTM was also 2 months (95% CI 1.42-2.58 months) for SD patients. This moment in time may be used as a benchmark for the incorporation of local therapy (33). Similarly, the median TTR for responders following TKI treatment is 7.4 weeks in Wu T et al.’s (40) trial. However, Jia W et al. (41) concluded that a temporal overlap of > 20 days between TKIs and TRT was an independent predictor of grade ≥2 radiation pneumonitis. This shows that a shorter time interval between overlaps may be safer. When radiotherapy combined with targeted therapy, the timing of radiotherapy is still lack of high-level evidence, which needs to be further explored by prospective researches.

It is worth noting that radiotherapy combined with TKIs increases the incidence of radiation pneumonitis. Previous trials suggested that when the first generation of TKIs combined with radiotherapy, the incidence of radiation pneumonitis was up to 40%, and grade ≥ 3 pneumonitis rate was as high as 20% (36). RECEL study also demonstrated radiation pneumonitis of grade ≥ 3 was 16.7% in EGFR-TKI combined with radiotherapy group (42). Previous studies have shown that higher lung V20 is associated with a higher radiation pneumonitis (43). The risk analysis of a trial suggested that the V20 > 22%, V30 > 17% was linked with an increase in radiation pneumonitis (44). However, in Jia W et al.’s (45) retrospective study, although the median V20 was 10.9% (range 6.3-28.1%), 63.6% and 45.4% of the patients treated with thoracic radiotherapy and Osimertinib developed radiation pneumonitis of grade ≥ 2 and grade ≥ 3, respectively. This suggests that when different generations of TKIs are combined with radiotherapy, the median V20 should be adjusted appropriately. However, Jia W et al.’s (45) study sample size is very small, only 11 patients, bias may affect the results of the study. A study by Zhu L et al. (46) (NCT04636593) is under way to evaluate the safety of thoracic radiotherapy combined with almonertinib. The primary endpoint is the incidence of grade ≥ 3radiation pneumonitis. We look forward to the results of this study.

## Brain Metastasis

Metastases to the brain are common in patients with NSCLC, individuals with BMs have a dismal prognosis with survival rates of less than 10% after one year despite treatment (47, 48). The prevalence of lung cancer with BMs has grown in recent years, owing to advancements in illness diagnosis and extracranial disease systemic treatment (49). Among these individuals, advanced EGFR-mutant or ALK-positive NSCLC patients had a significant cumulative risk (>70%) of BMs (50).

Effective central nervous system (CNS) penetration is crucial when systemic medication therapy is used to treat individuals with BMs (51). According to conventional wisdom, only lipophilic tiny molecules (Mr<400 Da) can pass through the normal blood brain barrier (BBB) *via* diffusion and other transport processes. The bulk of chemotherapeutic agents are hydrophilic macromolecules that cannot cross the BBB in the absence of carrier proteins. Additionally, several drug-resistant efflux pumps, like as P-glycoprotein (P-gp) and multidrug-resistant-associated proteins, are present on the capillary surface of the BBB, which can further impede drug entry into brain tissues (52). TKIs, the small compounds employed in targeted therapy, had an advantage over chemotherapeutic agents in terms of BBB penetration, demonstrating therapeutic results in patients with BMs (53, 54).

Despite their low molecular weight, TKIs still appeared to have a limited ability to permeate CSF (17). For example, first- and second-generation EGFR-TKIs have a low penetration rate into the CSF, often less than 10% (14). While osimertinib has a greater peak in the CSF than first- and second-generation EGFR-TKIs, its concentration in the CSF is still significantly lower than in plasma (15, 16).

To circumvent this constraint, various researches have advocated the inclusion of radiation. Brain irradiation has been shown to disrupt the BBB and improve the concentration of TKIs (17, 27). A prospective study (55) showed that the permeability of BBB increased after 2-4 weeks of treatment with WBRT (30 Gy/10f or 37.5 Gy/15f) or SRS (24, 18, or 15 Gy).

He ZY et al. (56) conducted a retrospective cohort analysis to compare the efficacy of concurrent EGFR-TKIs and whole brain radiotherapy (WBRT) against EGFR-TKIs alone in patients with advanced EGFR-mutant NSCLS with BMs. They evaluated the medical records of 104 treatment-naïve advanced NSCLC patients with EGFR mutantion and BMs, 56 patients received both EGFR-TKIs and WBRT, and 48 patients received just -TKIs. The researchers discovered that combining EGFR-TKIs and WBRT significantly increased median iPFS (17.7 vs 11.0 months, P=0.015) but did not significantly improve median OS (28.1 vs 24.0 months, P=0.756). Additionally, when compared to EGFR-TKIs alone, concurrent EGFR-TKIs with WBRT enhanced median iPFS in patients with more than three BMs (P=0.001); however, there was no significant difference in median iPFS between the two treatment regimens in patients with three or fewer BMs (P=0.526).

Slightly different from the results of the He ZY’s (56) study, Chen C et al. (57) observed that EGFR-TKIs combination with WBRT improved both iPFS (11.9 vs 10.2 months, p=0.039) and OS (21.0 vs 16.7 months, p=0.043) in NSCLC patients with EGFR mutations and BMs compared with just -TKIs. While the foregoing findings are intriguing, they are retrospective and need be further explored and confirmed in large-scale prospective clinical trials.

However, severe late toxicity often occurs 90 days after radiotherapy, including neurocognitive impairment, radiation necrosis and leukoencephalopathy (58). With the rapid development of stereotactic radiosurgery (SRS), radiotherapy provides an effective strategy for BMs. SRS for the intracranial oligometastatic disease has shown promising results. Compared with WBRT, SRS provides more focal and aggressive radiation, as well as more normal tissue protection (59).

Study by Yu F et al. (60) have shown that PFS (19.0 months vs 12.4 months, P=0.033) and OS (40.1 months vs 24.5 months, P=0.026) are significantly beneficial to patients with brain oligometastases (1 to 3 BM lesions with a maximal size of ≤3 cm) in upfront SRS combined with Osimertinib compared with Osimertinib alone. However, this study did not evaluate the treatment toxicity. NCT03535363 (phase 1) and NCT03769103 (phase 2) clinical trials are currently in the recruitment phase, which aims to research the efficacy and toxicity of SRS combined with Osimertinib in the treatment of BMs in patients with EGFR-mutant NSCLC. We look forward to the results of the study.

Regarding patients with ALK rearrangement, Takeda M et al. (61) stated that sustained administration of crizotinib following radiotherapy for isolated CNS advancement may be a treatment option, brain radiotherapy may also prolong the duration of crizotinib use. Similarly, Johung K et al.’s (62) retrospective analysis demonstrated that patients with NSCLC who had BMs and ALK- rearranged have an extended survival (OS: 49.5 months; iPFS: 11.9 months) when treated with radiation (SRS and/or WBRT) with TKIs.

In addition, Gadgeel S et al. (63) conducted a phase III ALEX study in patients with treatment-naïve stage IV ALK+ NSCLC, the result showed that compared with crizotinib, alectinib has better CNS activity, and patients with previously brain radiotherapy had higher intracranial objective response rate (86% vs 79%) and intracranial duration of response (not reached vs 17.3 months) compared with patients without previous radiotherapy.

Studies on the value of radiotherapy for NSCLC with BMs and EGFR or ALK mutation are summarized in **Table 2**.

**Table 2:** Clinical outcomes of TKI alone or combined with RT for BMs from NSCLC.

Study	Study type	Treatment Arm 1	NP	iPFS (months)	OS (months)	Treatment Arm 2	NP	iPFS (months)	OS (months)	P value
Welsh et al. (64)	prospective	–	–	–	–	erlotinib+WBRT	9	12.3	19.1	–
Chen et al. (65)	retrospective	TKI	79	18.2	41.1	WBRT+TKI	53	24.7	48	P_1 =_ 0.004 P_2 =_ 0.740
Johung et al. (62)	retrospective	–	–	–	–	RT+TKI	84	11.9	49.5	–
Fan et al. (66)	retrospective	TKI	41	13.9	27.9	RT+TKI	56	22.4	31.9	P_1 =_ 0.043 P_2 =_ 0.237
Zhu et al. (67)	retrospective	TKI	66	11.5	15	RT+TKI	67	16	22	P_1 =_ 0.017 P_2 =_ 0.015
Wang et al. (68)	meta-analysis	TKI	534	N	N	RT+TKI	534	N	N	–
He et al. (56)	retrospective	TKI	48	11	24	TKI+WBRT	56	17.7	28.1	P_1 =_ 0.015 P_2 =_ 0.756
Saida et al. (69)	retrospective	TKI	65	11	24	RT+TKI	39	15.6	26.1	P_1 =_ 0.096 P_2 =_ 0.525
Dong et al. (70)	meta-analysis	TKI	790	N	N	RT+TKI	763	N	N	–
Chen et al. (57)	retrospective	TKI	72	10.2	16.7	WBRT+TKI	76	11.9	21	P_1 =_ 0.039 P_2 =_ 0.043
Liu et al. (71)	retrospective	TKI	57	10.5	22.7	RT+TKI	77	18.9	30.8	P_1 =_ 0.0009 P_2 =_ 0.0183

NP, number of patients; N, no mention in the paper; RT, radiotherapy; WBRT, whole brain radio therapy; TKI, tyrosine kinase inhibitor; iPFS, intracranial progression-freesurvial; OS, overall survival; P_1_, P value of iPFS; P_2_, P value of OS.

About the intervention timing of radiotherapy for BMs, Magnuson WJ et al. (72, 73) discovered that early EGFR-TKIs and deferred radiotherapy were linked with a poor OS in EGFR-mutated NSCLC patients with BMs. Wang W (74) discovered that postponing brain radiotherapy may result in a shorter iPFS for EGFR-mutant NSCLC patients who have asymptomatic BMs. Chen H et al. (75) have indicated that treating patients with EGFR-TKIs and WBRT concurrently improves their short- and long-term outcomes compared to treating them sequentially or separately. Additionally, Miyawaki E et al. (76) reported that upfront local therapy resulted in a significantly improved OS and iPFS in patients with 1-4 BMs patients when compared to upfront TKIs, but no difference between the two groups in patients with ≥5 BMs. These findings suggest that commencing brain radiotherapy early may benefit survival.

Contrary to the above researches’s results, Liu S et al. (77) discovered that the timing of brain radiotherapy has no influence on OS for EGFR-mutant NSCLC patients who have asymptomatic BMs. The research of Chen C et al. (57) has the same result as that of Liu S et al. (77) nevertheless, they found that compared with patients in the latter radiotherapy, the iPFS and OS tend to be prolonged in patients who were treated with WBRT whether upfront or concurrent EGFR-TKIs, but there was no statistical difference in the result. Therefore, prospective researches into the best method for patients with EGFR-mutant NSCLC who have BMs is crucial, with a focus on the timing of local therapies and the amount of BMs.

## Bone Metastasis

Bone metastasis has a negative effect on the patient’s quality of life and is linked to a lower chance of survival. Around 80% of patients with bone metastatic suffer from pain, and more than 60% experience skeletal-related events such as bone surgery, bone radiotherapy, pathological fractures, spinal cord compression, and hypercalcemia (78). Previous trials have indicated that local irradiation for bone metastases might improve symptoms and perhaps prolong survival in patients with stage IV NSCLC who had an EGFR mutation (79, 80).

However, Hu F et al. (81) reported that the PFS and OS were not significantly different between the combination group and TKI group (monotherapy group) (PFS: 13.5 vs 10 months, P=0.175; OS: 33 vs 21 months, P=0.250) in lung adenocarcinoma patients with bone oligometasteses, despite an increasing trend in the local consolidative therapy (LCT) and TKI group (combination group). The tiny sample size could explain why statistical significance was not achieved.

In order to furtherly study whether local treatment can benefit the survival of patients with bone metastasis, Hu F et al. (82) expanded the study sample. 127 lung adenocarcinoma patients who had EGFR mutations and bone oligometastases were assessed. 65 patients got EGFR-TKIs alone (monotherapy group) and 62 patients got EGFR-TKIs in combination with LCT (combination group). As paired with monotherapy group, LCT was linked with meaningfully longer OS (36.3 vs 21.0 months, P=0.01) and PFS (14.0 vs 8.1 months, P=0.01). The findings corroborated previous researches, indicating that the combination group experienced meaningfully longer OS and PFS than the monotherapy group (83, 84). Thus, in patients with EGFR-mutated bone oligometastatic NSCLC, LCT in conjunction with EGFR-TKIs may be a preferable therapy option than monotherapy.

## Oligometastases

In 1995, Hellman S et al. (85) elucidated the concept of oligometastases, which consists of patients with metastases limited in number and organ sites (up to 3–5) who may have more indolent biology behavior. Similarly, a multidisciplinary European consensus recently classified oligometastatic illness as having five or fewer metastatic lesions involving no more than three separate organs. Lymph nodes in the mediastinum were not considered metastatic sites (86, 87). Earlier research revealed that oligometastasis was a distinct condition occurring between locally advanced stage and widespread stage IV (88).

Numerous researches have demonstrated that patients with these disease characteristics may benefit from aggressive local therapies in all metastatic and original lesions (83, 89). The newly published long-term findings of the SABR-COMET phase II randomised trial suggested that local ablative therapy increased the 5-year OS rate in oligometastatic patients to 42.3%, compared to 17.7% with standard of care therapies (90). However, no specific information about patients with oncogene driver mutations can be gleaned from this investigation.

SINDAS (91) is a multi-institutional, phase III clinical trial that is randomised, open label, and aims to assess the efficacy of radical local therapy in oligometastatic NSCLC. The study enrolled 133 patients, 65 patients in the TKIs group receiving TKIs alone and 68 patients in the stereotactic body radiotherapy (SBRT) group receiving TKIs combined with SBRT. After a median follow-up of 19.6 months, the median PFS was 12.5 months for TKIs group and 20.2 months for SBRT group, respectively (P<0.001). The median OS was 17.4 months in the TKIs group, whereas in the SBRT group was 25.5 months, respectively (P<0.001). Adverse events occurred similarly across groups. Additionally, Hu F et al. (81) and Xu Q et al. (92) found that compared to first-line TKIs alone, LCT improved PFS and OS.

In addition, Gan G et al. (93) advocated that SBRT for all oligometastatic foci in NSCLC patients with ALK rearrangement when the disease progressed during the treatment with crizotinib, reporting no grade 3-5 toxicity and a median, one-year, and two-year OS were 39 months, 86%, and 57%, respectively. The above-mentioned experimental results suggest that combining TKIs and radiation may be a viable treatment option for patients with oligometastatic oncogene driver-mutated NSCLC.

**Table 3** summarizes available clinical trials on patients with oligometastatic NSCLC who received local radiation to progressive focus.

**Table 3:** Selected studies of RT or SBRT treatment for oligoprogressive oncogene-driven NSCLC.

Study	Study type	Treatment Arm 1	NP	PFS (months)	OS (months)	Treatment Arm 2	NP	PFS (months)	OS (months)	P value
Xu et al. (92)	retrospective	TKI	51	13.9	30.8	LAT+TKI	39	20.6	40.9	P_1_<0.001 P_2_<0.001
Hu et al. (81)	retrospective	TKI	88	10	21	LCT+TKI	143	15	34	P_1 =_ 0.000 P_2 =_ 0.001
Wang et al. (91)	prospective	TKI	65	12.5	17.4	RT+TKI	68	20.2	25.5	P_1_<0.001 P_2_<0.001
Elamin et al. (30)	retrospective	TKI	129	14	–	RT+TKI	12	36	–	P_1 =_ 0.0024
Arrieta et al. (94)	prospective	–	–	–	–	LAT +TKI	16	17.9	not reached	–
Weiss et al. (95)	prospective	–	–	–	–	SBRT+TKI	25	6	29	–

EGFR, epidermal growth factor receptor; ALK, anaplastic lymphoma kinase; RT, radio therapy; SBRT, stereotactic bodyradio therapy; N, no mention in the paper; PFS, progression-freesurvial; OS, overall survival; LAT, local ablative therapy; LCT, local consolidative therapy; P_1_, P value of PFS; P_2_, P value of OS.

## Elderly Patients

Considering the general condition and associated diseases of elderly patients, targeted therapy may be more suitable for elderly patients with advanced NSCLC with oncogene driven mutations compared with traditional chemotherapy. Kashiwabara K et al.’s (96) study suggested that in patients (aged≥85 years) with a PS score of 0-2, compared with the chemotherapy group or the best supportive care (BSC) group, the OS of the TKI group tended to be longer (16.9 months vs.7.2 months or 9.8 months, P=0.059), and did not increase the incidence of serious adverse events. Yamada Y et al.’s (97) study indicate that in elderly patients (over 75 years of age) with advanced NSCLC and EGFR mutations, EGFR-TKI rechallenge after first-line EGFR-TKI treatment was effective and tolerable.

Does the addition of radiotherapy improve the survival of elderly NSCLC patients with oncogene driver-mutation? A Chinese study (98) included 122 elderly NSCLC patients who treated with gefifitinib and SBRT. The patients were divided into three groups: group A (gefifitinib + SBRT), group B (SBRT alone) and group C (gefifitinib alone). The results showed that compared with group B and C, group A had the benefits of PFS (7.8 vs 5.9, P=0.018 and 7.8 vs 5.1, P=0.013, respectively) and OS (15.5 vs 9.6, P=0.002 and 15.5 vs 10.3, P=0.017, respectively). There are few studies on radiotherapy combined with targeted therapy in elderly NSCLC patients with oncogene driver-mutation, and large-scale randomized controlled trials are needed to explore the survival benefits of radiotherapy for these patients.

## Conclusions

The therapy of NSCLC has changed dramatically as a result of recent molecular biology advances. TKIs marked a turning point in the diagnosis and treatment of advanced oncogene driver-mutated NSCLC patients. TKIs’ long-term efficacy, nevertheless, is hampered by acquired drug resistance. In comparison to TKIs alone, a combination of anti-EGFR or anti-ALK medicines and radiation has been shown in numerous clinical studies to improve survival outcomes. Several aspects maybe attributed to the improvement. The radiotherapy reduced TKIs resistance and prolonged the treatment of the targeted therapy and TKIs also radiosensitized the treatment of radiotherapy. Therefore, the combination of targeted treatments and radiation is an encouraging and promising strategy for advanced oncogene driver-mutated NSCLC. However, several challenges still lie ahead. The optimal timing of radiation and the techniques to be used have not yet been determined. The best method of TKIs-radiotherapy combination in the treatment of advanced oncogene driver-mutated NSCLC requires to be further explored by large-scale prospective studies. In recent years, immunotherapy is on the rise, however, the effect of first-line immunotherapy is poor for NSCLC patients with EGFR mutation or ALK rearrangement. For these patients after targeted treatment of drug resistance, whether the addition of immunotherapy will bring survival benefits is worthy of further study.

## Author Contributions

SY conceived and supervised the study. JC consulted the literature and wrote the manuscript. LL provided critical revision. All authors contributed to the article and approved the submitted version.

## Funding

This study was partially supported by the National Natural Science Foundation of China (grant No. NSFC81872475 and NSFC82073345) and Jinan Clinical Medicine Science and Technology Innovation Plan (202019060) to SY.

## Conflict of Interest

The authors declare that the research was conducted in the absence of any commercial or financial relationships that could be construed as a potential conflict of interest.

## Publisher’s Note

All claims expressed in this article are solely those of the authors and do not necessarily represent those of their affiliated organizations, or those of the publisher, the editors and the reviewers. Any product that may be evaluated in this article, or claim that may be made by its manufacturer, is not guaranteed or endorsed by the publisher.
